# Comparative analysis of remotely-sensed data products via ecological niche modeling of avian influenza case occurrences in Middle Eastern poultry

**DOI:** 10.1186/1476-072X-10-21

**Published:** 2011-03-28

**Authors:** Sarah Bodbyl-Roels, A Townsend Peterson, Xiangming Xiao

**Affiliations:** 1 Biodiversity Institute and Department of Ecology and Evolutionary Biology, The University of Kansas, Lawrence, Kansas 66045 USA; 2 Center for Spatial Analysis, Department of Botany and Microbiology, University of Oklahoma, Norman, Oklahoma 73019-5300, USA

## Abstract

**Background:**

Ecological niche modeling integrates known sites of occurrence of species or phenomena with data on environmental variation across landscapes to infer environmental spaces potentially inhabited (i.e., the ecological niche) to generate predictive maps of potential distributions in geographic space. Key inputs to this process include raster data layers characterizing spatial variation in environmental parameters, such as vegetation indices from remotely sensed satellite imagery. The extent to which ecological niche models reflect real-world distributions depends on a number of factors, but an obvious concern is the quality and content of the environmental data layers.

**Methods:**

We assessed ecological niche model predictions of H5N1 avian flu presence quantitatively within and among four geographic regions, based on models incorporating two means of summarizing three vegetation indices derived from the MODIS satellite. We evaluated our models for predictive ability using partial ROC analysis and GLM ANOVA to compare performance among indices and regions.

**Results:**

We found correlations between vegetation indices to be high, such that they contain information that overlaps broadly. Neither the type of vegetation index used nor method of summary affected model performance significantly. However, the degree to which model predictions had to be transferred (i.e., projected onto landscapes and conditions not represented on the landscape of training) impacted predictive strength greatly (within-region model predictions far out-performed models projected among regions).

**Conclusion:**

Our results provide the first quantitative tests of most appropriate uses of different remotely sensed data sets in ecological niche modeling applications. While our testing did not result in a decisive "best" index product or means of summarizing indices, it emphasizes the need for careful evaluation of products used in modeling (e.g. matching temporal dimensions and spatial resolution) for optimum performance, instead of simple reliance on large numbers of data layers.

## Background

Ecological niche modeling and the associated species distribution modeling (here referred to collectively as 'niche modeling' for convenience) are techniques in which occurrence data for biological phenomena are related to raster geospatial data sets describing relevant aspects of environmental landscapes to estimate species' ecological requirements and potential geographic distributions [1-3]. Much of the burgeoning activity in this field has based inferences on climate data [4], yet climate data have severe limitations: (1) high spatial autocorrelation in most climate features limits the spatial resolution possible in inferences from such data sets, (2) climate data do not reflect land use and land cover (directly, at least) that provide important refinements to distributional predictions [5], (3) climate data provide only very limited ability to build time-specific elements into such models [6,7], and (4) climate data for certain regions and spatial resolutions will be mostly interpolation rather than real information [4]. As a consequence, researchers have begun using remotely sensed data in these exercises [8,9]--remotely sensed data offer the potential for analyses at fine spatial resolutions, incorporating non-climatic dimensions into models, and achieving excellent temporal resolution simultaneously.

**Figure 1:**
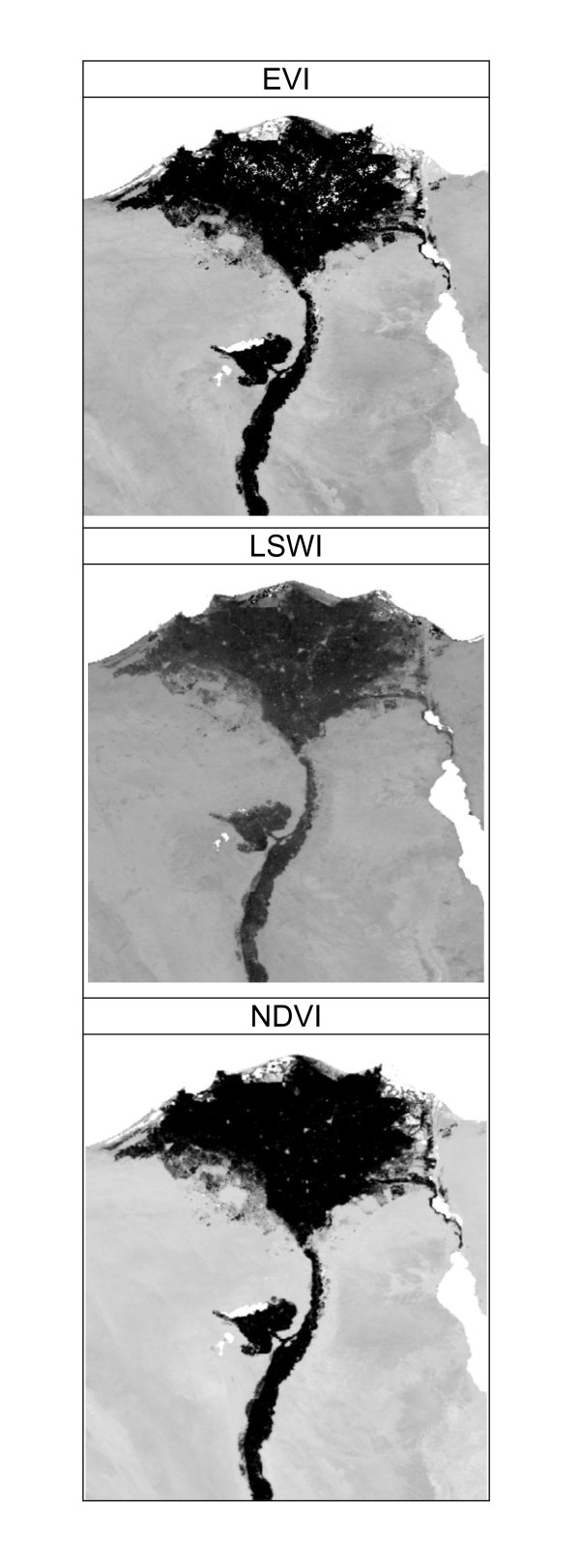
**Detail of the Nile Delta in January 2005, to illustrate patterns reflected in values of the three remotely sensed indices explored in this study**. The gray scale illustrates gradients from low (white) to high (black) values of each index. (a) Enhanced Vegetation Index, (b) Leaf Surface Water Index, (c) Normalized Difference Vegetation Index.

Initial exploratory use of remotely sensed data in niche modeling, however, has incorporated remotely-sensed information rather uncritically. Some researchers have used multi-temporal vegetation index values to characterize landscapes [e.g., [10]], whereas others have used summary statistics such as means and variances or more complex summaries of seasonality and environmental variability [11]; the relative efficacy of these different approaches, however, has not been assessed quantitatively. Similarly, although numerous indices and distinct remotely sensed data products have been used (e.g., AVHRR, MODIS, and Landsat imagery; NDVI, LSWI, EVI and other indices; see examples in Figure 1), no detailed comparisons of the performance of the resulting niche models have been presented. Perhaps the most detailed treatment of such data sets to date used Fourier transforms to extract signals of seasonality on global scales [11], which sounds quite promising for improving such applications, but neither the algorithms nor the resulting data have been made openly available (A. T. Peterson to authors, in litt., 2008, 2009). In general, although remotely-sensed data sets have seen increasing use in niche modeling, they have not seen adequate testing or evaluation to establish which data sets and in which combinations will prove most robust and adequate for such applications.

The purpose of this contribution is to develop a comparative analysis of six remotely-sensed data sets for models of the distribution of avian influenza cases, largely in domestic poultry, in the Middle East and surrounding areas. Although these veterinary cases affect only domestic birds, the single highest-ranked risk factor in all studies to date of H5N1 transmission has been contact with (infected) poultry [12-14], so understanding spatial risk in poultry infections has direct and immediate implications for human infections. Avian influenza occurrences in this region have already been reported in detailed analyses [15], but without careful evaluation of the relative merits of the different remotely-sensed data products that may be used for input. Here, we compare and contrast predictions within and among four sectors of the Middle East based on models incorporating three vegetation indices derived from image data from the MODIS sensor, each in the form of monthly values versus summary variables. The result is a first detailed view of situations under which remotely sensed data can be used most appropriately in niche modeling applications.

## Methods

### Study Region

**Figure 2:**
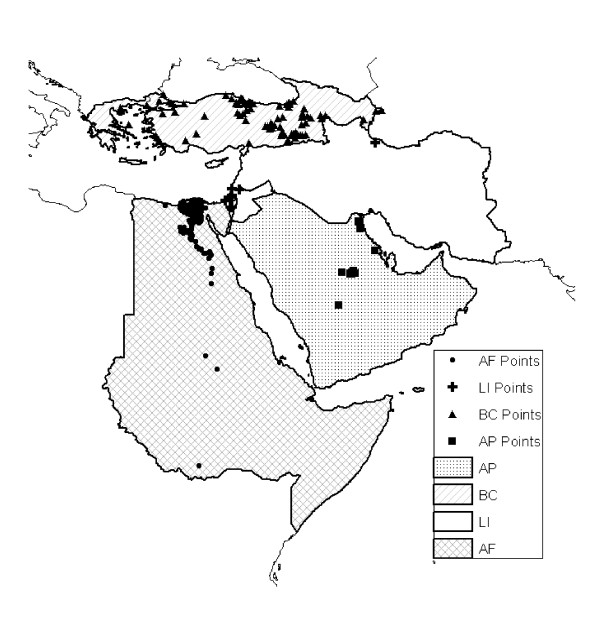
**Summary of four subregions of the Middle East across which models were developed and predictions tested: AF = northeastern Africa, BC = the Balkans and Caucasus region, LI = the Levant and Iran, and AP = the Arabian Peninsula**. Occurrence points for H5N1 avian influenza are shown as dark symbols.

Model tests were based on the four spatial subsets of the Middle East and northeastern Africa (Figure 2) that were originally analyzed in an earlier contribution [15]. These subregions were chosen based on spatial contiguity, reporting characteristics and consistency, and numbers of avian influenza case occurrence rates in poultry. We used these four spatial subsets to partition the overall region for testing the predictive ability of models.

### Environmental Datasets

Environmental datasets consisting of 12 monthly composite remotely-sensed data layers for 2005 were assembled, summarizing maximum values for 8-day time series of the Enhanced Vegetation Index (EVI), Normalized Difference Vegetation Index (NDVI), and Land Surface Water Index (LSWI; see Figure 1 for example comparisons). Layers were derived from the Moderate Resolution Imaging Spectroradiometer (MODIS) satellite-based sensor at a spatial resolution of 500 m. Resolution for all layers was coarsened subsequently to a 5 km resolution, via nearest-neighbor resampling, owing to computational limitations and concerns regarding the spatial accuracy of the occurrence data. Summary statistics layers, including maximum, mean, minimum, range, and standard deviation across the 12-month period, were produced for each index, resulting in 17 raster data layers (12 monthly summaries and 5 summary statistics) for each index (EVI, LSWI, NDVI). Owing to concerns regarding the effects of snow cover on the EVI and LSWI indices [16], we also conducted limited tests for EVI-based models trained in the Balkans-Caucasus region based on only the snow-free months to see whether excluding months with snow cover would improve model predictivity.

### Occurrence Points

Geographic coordinates for 610 spatially unique case occurrences of highly pathogenic avian influenza of the H5N1 strain (31 from the Arabian Peninsula AP, 18 from the Levant-Iran LI, 386 from northeastern Africa AF, and 175 from the Balkans-Caucasus BC regions) were drawn from the World Organization for Animal Health [17] databases as part of a previous study [15]. These case-occurrences included virus isolations from wild birds, zoo birds, and commercial and backyard poultry.

### Ecological Niche Modeling

Ecological niches were estimated using the Genetic Algorithm for Rule-set Prediction [GARP; [18]], an evolutionary-computing algorithm that has been applied widely to questions of disease transmission [19]. GARP's predictive ability has been tested under diverse circumstances [3,20,21] and has been demonstrated to yield highly predictive hypotheses similar to those produced by other methods [e.g., Maxent; [22]]. GARP has been the basis for both of our previous studies of avian influenza transmission ecology and geography, showing highly statistically significant predictive ability [15,23].

Within GARP processing, input occurrence data are divided randomly into three subsets: training data (25%; for model rule development), intrinsic testing data (25%; for intrinsic evaluation and tuning of model rules) and extrinsic testing data (50%; for evaluation of model quality and filtering among replicate models, see below). Spatial predictions of presence versus absence can include two types of error: false negatives (areas of actual presence predicted absent) and false positives [areas of actual absence predicted present; [24]]; rule performance in terms of overall error is evaluated via the intrinsic data set. Changes in predictive accuracy from one iteration to the next are used to evaluate whether particular rules should be incorporated into the model or not, and the algorithm runs either 1000 iterations or until convergence [18]. The final rule set is then used to query the environmental data sets across the study region to identify areas fitting the rule-set prediction, producing a hypothesis of the potential geographic distribution of the species [25], or in this case, of the potential area for disease transmission.

Since GARP processing includes several random-walk components, each replicate model run produces distinct results, representing alternative solutions to the optimization challenge. Consequently, following recommended best-practices approaches [21], we developed 100 replicate versions of each model. We filtered these replicates based on their error characteristics to emphasize the overriding importance of omission error (as opposed to commission error), retaining the 20 models showing the lowest false-negative rates (= percentage of independent testing points falling in areas not predicted to be suitable), and then retaining the 10 (of the 20) closest to the median of the proportional area predicted present among models, an index of false-positive error rates [21]. A consensus of these 'best subset' models was then developed by summing values for each pixel in the map to produce final predictions of potential distributions with 11 thresholds (i.e., integers from 0 to 10).

### Model Testing

Our aim throughout this contribution is to develop robust comparisons of model performance based on different remotely sensed data products (EVI, NDVI, LSWI) summarized in different ways (monthly values, summary statistics) across four regional datasets. The predictive challenge was to anticipate the spatial distribution of H5N1 avian influenza case-occurrence data within and among the four study regions in the Middle East and northeastern Africa. Hence, we created GARP models for all possible combinations of the following categories of occurrence and environmental data:

*• Index category*: To evaluate performance of different MODIS satellite data product types and effects on model predictivity, models were run based on environmental data sets of EVI, NDVI, LSWI, or the combination of all three.

*• Index type*: To evaluate model performance based on monthly index data *versus *summary data, models were created based on either the 12 monthly values of each index, or on the 5 summary statistics for each index, as well as on all 17 datasets available for each index.

*• Modeling level*: An important additional dimension is whether model predictions are simply interpolating among closely approximated training occurrence points, or whether they are being 'transferred' to other landscapes, on which sampling may be scanty or nonexistent [20]. Hence, we developed the following: (1) four analyses within single regions (i.e., training and testing data randomly selected from the same region, using only data from that region), (2) four in which three regions predict one (i.e., training based on occurrence points from three regions and projecting onto and validation in the fourth), and (3) four in which one region predicts three (i.e., training models within one region and projecting models onto and validation in the other three regions),

This modeling scheme thus resulted in a total of 4 × 3 × 12 = 144 GARP runs, summarized in Additional file 1: Appendix 1 and Additional file 2: Appendix 2.

Because our models are based on presence-only data, and because information documenting absences of suitability for influenza transmission across the Middle East are unavailable, customary approaches to model validation (e.g., receiver operating characteristic, kappa statistics) are neither appropriate nor applicable. As a consequence, we modified the receiver operating characteristic (ROC) approach so as not to depend on absence data by recasting the (1 - specificity) axis as the proportional area predicted present following Phillips et al. [26] and Peterson et al. [22]. The area under the curve (AUC) of traditional ROC approaches undervalues models that do not provide predictions across the entire spectrum of proportional areas in the study area [22]; in addition, traditional ROC approaches incorrectly weight the two error components (omission and commission) equally [22]. As a consequence, we used a modification of ROC that remedies these problems, a partial-area ROC approach that evaluates only over the range of the prediction, and potentially allows for differential weighting of the two error components [22].

We performed partial ROC analyses for each of the 144 model predictions, based on the independent sets of testing points not used to train the models. We calculated these values using a program developed by N. Barve and colleagues (pers. comm.; available upon request from the authors); we present our results as the ratio of the model AUC to the null expectation [AUC ratio; [22]]. AUC ratios were limited to the proportional area over which models made predictions, and expected error parameters of 0% (E = 100, equivalent to traditional ROC analyses) and 5% [a more appropriate parameter value for niche modeling applications; [22]] were both calculated for comparison. Bootstrapping to evaluate the statistical significance of AUC ratios compared to the null was performed by resampling 50% of the test points with replacement 1000 times from the overall pool of testing data; one-tailed probabilities associated with AUC ratios were assessed by counting numbers of bootstrap replicates with AUC ratios less than 1 (hereafter referred to as P, [Table 1, Additional file 1: Appendix 1, and Additional file 2: Appendix 2]). AUC ratios and P were explored further using general linear model (GLM) analysis of variance (ANOVA) and *post-hoc *Tukey-Kramer tests, using the statistical software package Minitab 14^®^.

**Table 1:** Summary of General Linear Model ANOVAS in this study.

		E = 100		E = 5%	
		
Test Variable	Random variables	AUC ratio	P	AUC ratio	P
Index	Test region	1.00 ns	0.51 ns	1.36 ns	2.28 ns
Data type	Test region	0.03 ns	0.38 ns	1.97 ns	0.69 ns
Training region (all)	None	1.23 ns	1.33 ns	3.87 **	2.47*
Training and testing region (within)	None	14.86**	4.97**	55.35**	6.90**
Testing region (all)	None	8.02**	7.57**	4.80**	6.11**
Model Level (within region, three predict one, one predict three)	None	23.02**	7.76**	8.68**	5.93**

F-values are reported for the test variable only. * denotes significance at the α = 0.5 level, ** denotes significance at α ≤ 0.01; ns = non-significant. "Index" indicates EVI, LSWI, and NDVI; "data type" indicates monthly *versus *summary data; "training region" and "testing region" refer to the 4 regions, analyzed either based on performance in all analyses ("all") or only in within-region analyses ("within"); and "model level" refers to numbers of regions predicting *versus *being predicted.

We compared predicted occurrence values (i.e., GARP-derived suitability thresholds ranging 0-10) from different modeling exercises to establish how consistent within-region patterns are with broader patterns of ecological associations. Specifically, we compared within-region-trained model predictions to values predicted for the same region by models trained based on the three *other *regions--this comparison illuminates the uniqueness of the occurrence-environment association for particular regions. We used fuzzy map comparison approaches to evaluate overall correspondence, via the Fuzzy Inference System in the Map Comparison Kit [MCK; [27]], which accounts for possible imprecision in correspondence among maps--pixel shifts over minor spatial extents may exist, which would confuse methods based on exact matching, but which are accounted for in fuzzy comparisons. Fuzzy comparisons were made for summary index types only, resulting in 4 index categories × 4 regions = 16 comparisons (Table 2).

**Table 2:** Fuzzy Interpolation values of model performance comparing predicted occurrence values (GARP thresholds 0-10) of within region models (occurrence points within region) to values predicted for the same region by models produced using the three other regions (occurrence points outside of region predicted).

Region	Index	Fuzzy kappa value
AF	LSWI	0.229
AF	EVI	0.472
AF	NDVI	0.225
AF	LSWI, EVI, NDVI	0.275
LI	LSWI	0.279
LI	EVI	0.175
LI	NDVI	0.381
LI	LSWI, EVI, NDVI	0.190
BC	LSWI	0.325
BC	EVI	0.288
BC	NDVI	0.362
BC	LSWI, EVI, NDVI	0.316
AP	LSWI	0.276
AP	EVI	0.596
AP	NDVI	0.410
AP	LSWI, EVI, NDVI	0.407

To test whether snow cover altered EVI values enough to bias model predictions, we re-ran 8 models (three regions predict one, monthly and monthly + summary statistics) where AF, LI, and AP predicted occurrences in BC. Because the BC region receives the most seasonal snowfall of our four regions, testing models using BC points would be most likely to reveal any discrepancies owing to snow biased EVI values. Environmental data from months November to February, when BC receives the most snow, were removed from model construction. Paired t-tests comparing AUC ratios and P values at E = 100 and 5% including and excluding winter months resulted in all non-significant test values, thus we did not consider our EVI data to be biased markedly by snowfall, and used complete 12-month models for all further analyses.

Finally, we explored the degree to which the different MODIS satellite indices provide independent sets of information, as opposed to highly correlated and redundant information. To examine this possibility, we determined Pearson product-moment correlations for each pairwise combination of the three indices (EVI, LSWI, NDVI) based on the five summary statistics (max, mean, min, range, standard deviation) calculated for each of the four study regions. We generated 10,000 random points within each region, and extracted index values for each statistic at each point in ArcGIS (version 9.2), resulting in 3 indices × 5 summary statistics × 4 regions = 60 comparisons. Correlations were calculated from these data using Minitab 14^®^.

## Results

Model building and testing was based on four geographic regions and 610 occurrence data points divided variously into different training and testing sets (Figure 2). Sampling was least dense in the Levant-Iran (LI) region (only 18 occurrence points), with the greatest concentration of points within this region in Israel. The northeastern Africa (AF) region had the most occurrence points (386), with the vast majority clumped along the Nile River and its delta. Sampling was most widespread and even across the Balkans-Caucasus region (175), with points distributed across multiple countries.

In all, 144 niche models were developed for this study. The associated tests of predictive ability indicated that independent testing points often coincided with model predictions considerably better than random expectations. In all, 70 model predictions were significantly more coincident with independent testing data (bootstrap value, P < 50; see Additional file 1: Appendix 1 and Additional file 2: Appendix 2) when no error considerations were weighed (i.e., E = 100). A slightly better 81 model predictions were significantly better than random expectations when some nonzero expectation of error was included (E = 5%). A paired t-test for differences between all model AUC ratios when E = 100 (mean 1.16) and 5% (mean 1.06) was significant (T = 7.01, *p *< 0.001), indicating that model fit improved with the error consideration. An identical test for P when E = 100 (mean 258) and 5% (mean 200) was also significant (T = 2.62 *p *= 0.01). E = 5% also reduced the variance in AUC ratios (Levene's test for equal variances, L = 28.93, *p *< 0.005) but not in P (L = 1.88, *p *= 0.171).

### Within Regions

**Figure 3:**
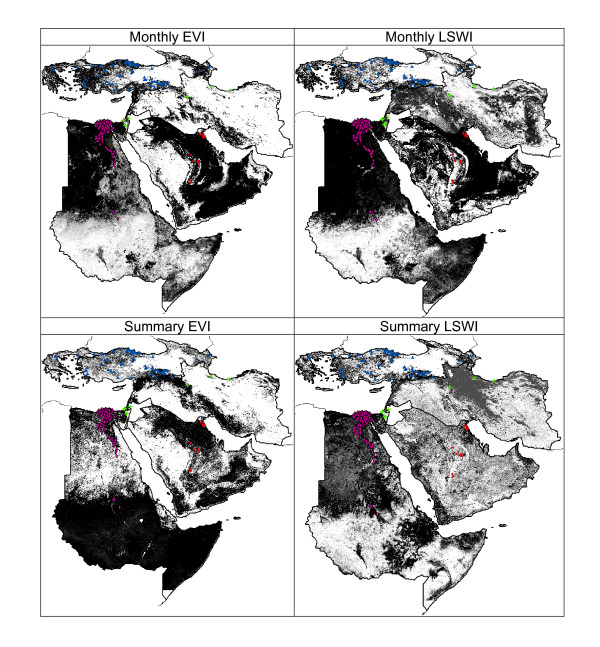
**Within-region model predictions of H5N1 avian influenza case occurrences for monthly and summary statistic index datasets, and for Enhanced Vegetation Index and Leaf Surface Water Index**. Shading indicates model agreement in predictions of presence: white = all models predict absence to black (all models predict potential for presence). In each map, predictions from the four within-region predictions are mosaicked. Data used in testing models are shown for each region within the mosaics.

These tests evaluated the ability of models to predict H5N1 case occurrences based on subsets of known occurrence points within regions based on different remotely sensed data products. Models based on all combinations of index and data type resulted in 16 models and associated tests, producing reasonably similar patterns of predictivity (Figure 3). Validation tests based on independent sets of occurrence points concluded that model fit was generally good (Additional file 1: Appendix 1): 63% of model predictions were significantly better than random for E = 100, and 56% were significant for E = 5% (Additional file 2: Appendix 2).

Model fit varied widely among regions. AF was the region that showed the best predictivity, as 100% of tests were highly significant (all bootstrap values P < 50, regardless of E). However, AF also showed great variation in patterns of spatial suitability predicted. For example, a model based on summary EVI data predicted high suitability in southern Sudan, Ethiopia, and Somalia, while other models predicted high suitability only in the northern portion of the region (northern Sudan and Egypt; see Figure 3). Regions AP, BC, and LI were more variable in predictive ability, with 50%, 31%, and 22% of models resulting in significant predictions, respectively.

### Three Regions Predicting One

These tests evaluated the predictive success of models based on occurrence points from three regions in the challenge of predicting suitability for H5N1 case occurrences in the fourth region; hence, the model was tested with independent occurrence points from that region. Because of the relative spatial independence of the training vs. testing points, predictive success in these tests was lower as compared with those of within region predictions: 35% showed significant predictive ability when E = 100, and 56% when E = 5% (Additional file 2: Appendix 2).

**Figure 4:**
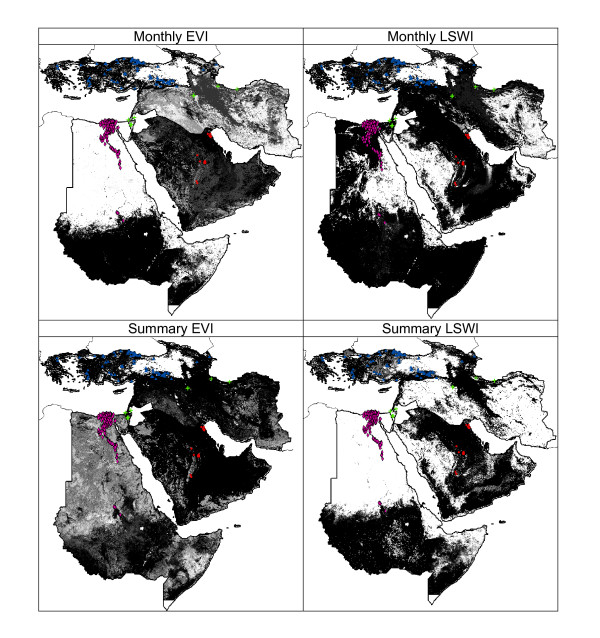
**Among-region model predictions of avian influenza case occurrences for monthly and summary statistic index datasets, and for Enhanced Vegetation Index and Leaf Surface Water Index, in which occurrence data from three regions are used to predict the distribution of case occurrences in the fourth region**. Shading indicates model agreement in predictions of presence: white = all models predict absence to black (all models predict potential for presence). In each map, predictions from the four regional predictions are mosaicked. Data used in testing models are shown for each region within the mosaics.

Again, model fit varied among regions: AP and AF had the greatest number of models significant, with 75% and 58% of tests showing significance, respectively. LI tests were 46% significant and BC was least predictive, with only one test (4%) showing predictivity better than random expectations. Geographic regions predicted as suitable for H5N1 case occurrences were also more diverse across types of model input than within region predictions (Figure 4). For example, in AF, models varied widely in predicting suitability restricted either to the vicinity of the Nile River or throughout surrounding areas (Figure 4). However, some regions were predicted as suitable consistently by all of the models: the Nile River and its delta, southern Somalia, coastal eastern Saudi Arabia, central Iran, and coastal western Turkey (Figure 4). Despite this general model agreement, these spatially stratified model predictions did not match well with those of within-region models: fuzzy pattern matching values evaluating pattern agreement among the two suites of models were all below 0.6, while close agreement is considered as values above 0.7 [Table 2; [27]].

### One Region Predicts Three

This modeling challenge was clearly the most difficult: predicting spatial patterns of suitability across three regions based on independent occurrence points in the fourth. Twelve models were created for each set of three regions utilizing the previous combinations of index and data type (Additional file 1: Appendix 1; results not shown). For E = 100, 48% of model predictions were significantly better than random expectations, while 56% were significantly better than random at E = 5% (Additional file 2: Appendix 2). The region from which models were best able to predict suitability across the other three was BC, with 83% of models significant, while the one least able to predict regional suitability was AF, with only 21% of models significant. We suspect that this variation in predictive ability reflects the degree to which the environments in the model training region are representative of the environments in the testing region, eliminating the need for model clamping [26].

Regions predicted as suitable for H5N1 varied immensely, highly dependent upon the region used to calibrate models. For example, when AF modeled occurrence in LI, BC, and AP, the most suitable regions included the Fertile Crescent and the deserts of southwest Iraq, eastern and southern Saudi Arabia, Yemen, and Oman. In contrast, LI predicted occurrence in the opposite regions of BC, AP and AF, such as southern Iran and the eastern coast of the Red Sea. Most models, however, reached a consensus that the most likely areas for H5N1 suitability include the Nile River and its delta, southwest Somalia, and the coastal region along the Azir mountains of Saudi Arabia and the Sarawat mountains of Yemen.

### Overall Trends

Analyses of model performance across all index categories, index types, and modeling levels produced interesting results (Table 1). First, no difference in model performance was detectable among indices (ANOVA, F = 1.00, p = 0.396, when E = 100, F = 1.36, p = 0.258, when E = 5%). We also found no difference in model performance between monthly and summary index datasets (ANOVA, F = 0.03, p = 0.974, when E = 100, F = 1.97, p = 0.143, when E = 5%). This lack of significant difference likely indicates that correlations across the study area are high among the different environmental data sets, and that each provides little independent information, at least at this broad spatial extent.

**Figure 5:**
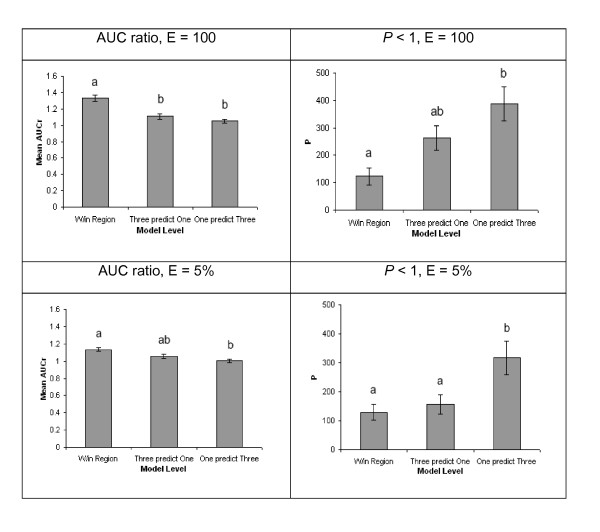
**Means and standard errors for all four test statistics (AUC ratio and P at E = 100, 5%) by modeling level**. Bars labeled with unique letters denote significant differences between categories (Tukey-Kramer post-hoc tests).

**Figure 6:**
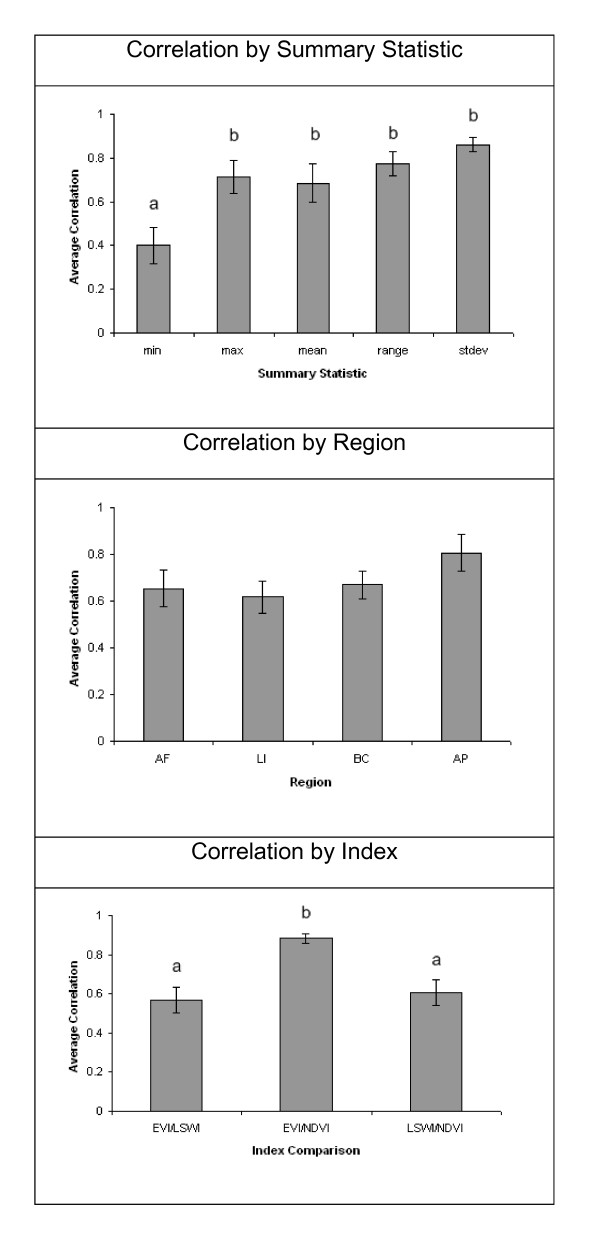
**Means and standard errors of correlations among data points by index types, based on index values at 10,000 random geographic points across the study region**. Panel (a) shows differences in mean index correlation by summary statistic. Panel (b) shows no difference in index correlation among study regions. Panel (c) show that indices EVI and NDVI are more similar to one another than EVI/LSWI or NDVI/LSWI. Bars labeled with unique letters denote significant differences between categories (Tukey-Kramer post-hoc tests).

We did, however, observe significant differences in predictive ability of models among regions (Table 1). Predictive ability differed among regions tested intrinsically with random samples of occurrence points and in spatially-stratified predictions among regions (Table 1). Regional differences were also noted (ANOVA, all *p *≤ 0.003) in tests of effects of modeling level (i.e., prediction within regions, three regions predict one, one region predicts three). *Post hoc *tests on each test variable (AUC ratios and P when E = 100, 5%) showed pervasive differences among mean values between predictions within regions and one region predicting three (Figure 5). By region, Pearson correlation coefficient values (not shown) for each pairwise combination of indices (EVI, LSWI, NDVI) by each of the five summary statistics averaged 0.687 (range, 0.052 to 0.984). There were no observed differences in index correlation among regions (Figure 6b, ANOVA F = 1.33, *p *= 0.273). Significant differences were apparent in correlation values among the summary statistics (Figure 6a, ANOVA F = 6.18, *p *< 0.001). Minimum values compared across index types over all four regions were less correlated than the other 4 summary statistics. High correlations between indices were also observed (Figure 6c): index types EVI and NDVI, both measures of photosynthetic mass, were consistently more similar to each other than any of the other index pairs (ANOVA F = 9.72, *p *< 0.001).

## Discussion

This study presents a first detailed comparative analysis of different remotely sensed data products as input information into ecological niche models. Our analyses are based on a spatially stratified model evaluation strategy, in which different geographic regions are used to train models and test model predictions. Furthermore, our use of the partial ROC approach allows considerably greater confidence in the model evaluation statistics, as it avoids several of the artifactual complications inherent in traditional implementations of ROC testing [22].

Although our analyses are based on veterinary cases, previous analyses of risk factors associated with H5N1 transmission have indicated an intimate tie between poultry infections and human cases [12-14], so the models presented here offer a view into human transmission risk not otherwise available in the Middle East region. Our results indicate that such data can provide a rich basis for modeling environmental correlates of occurrence (more or less equivalent to an ecological niche), and offer significant advantages in terms of spatial and temporal resolution, information content, and lack of need for interpolation. As such, we interpret the results of this study as confirming broadly and elucidating the role of remote sensing data in niche modeling exercises, which offered spatial predictions that were frequently much more informative than random expectations.

Our results could be interpreted as equivocal, in that we did not detect significant differences among vegetation index categories and vegetation index types in predictive ability of models. This non-effect could result from several factors. Among the artifactual causes that should be considered is the possibility that the avian influenza case occurrence data on which we based the tests could simply be too "noisy" to permit successful niche model development. Although the avian influenza data are far from perfect, they have nonetheless supported two detailed niche modeling exercises in the past [15,23] with considerable success.

An alternative explanation, and one that we favor, is that the information essential to the different vegetation indices is--in the end--derived from the same remotely sensed images, and in many cases from the same actual bands of data. In this sense, while the different vegetation indices (NDVI, EVI, LSWI, etc.) are not equivalent, the key information in one may overlap very broadly with that in another. NDVI, EVI and LSWI are related to leaf area index, canopy chlorophyll content, and water content, respectively [16]; these vegetation properties are closely related to each other. In the case of large-scale modeling at relatively coarse spatial resolution (e.g., 5-km gridcell in this study) we suspect that the important information in these vegetation indices may be highly correlated, such that one does not offer significant and consistent advantages over any other. Finally, in spite of known complications in EVI calculations for images in which snow cover is present [16], we found no marked improvement of models built in seasons lacking snow cover, but use of EVI is probably best restricted to times and places lacking snow cover nonetheless, in other words, within the plant growing season.

Perhaps the clearest and most important insight from our analyses is the trends in predictive success manifested as a negative relationship between model predictivity and the degree of transferability required of the model. That is, for within-region predictions, model performance was quite good, but for three-regions-predict-one-region and one-region-predicts-three-regions, the model predictions were successively less robust. In essence, spatial autocorrelations make the within-region tests relatively easy, while cross-regional transfers of model predictions are more difficult; in the worst cases, these predictions must transfer into regions with environments little-represented in the training region, thus introducing an element of extrapolation into predictions, a situation in which models are not likely to be robust. We noted, perhaps more than in other applications, a fair amount of variation among models as to the spatial location of areas predicted as highly suitable for H5N1 transmission--as such, we recommend development of large numbers of models and seeking of broad central tendencies, rather than interpreting the details of single or small sets of models.

## Conclusions

In sum, this paper illustrates a variety of points regarding the use of remote sensing data as data inputs to ecological niche models (or the related species distribution models, for that matter). While our testing did not detect a "best" index product or means of summarizing indices, it points to the need for careful and detailed understanding of patterns of intercorrelation and nonindependence among these data products, such that "quality" trumps "quantity," and the clear focus should be on correct matching of temporal dimensions, spatial resolution, and other qualities, rather than on inclusion of huge numbers of data layers in analyses. We hope that this paper will stimulate others to incorporate these datasets into their research and modeling endeavors, as they provide a rich and detailed basis for such models.

## Competing interests

The authors declare that they have no competing interests.

## Authors' contributions

ATP and XX conceived of the project. XX provided the raw satellite data. SBR carried out the modeling and analyses. ATP and SBR wrote the manuscript. All authors read and approved the final manuscript.

## Supplementary Material

Additional file 1 **Appendix 1: Summary of models developed and partial ROC test results**. Each of the 144 models developed are displayed along with associated AUC and P values for error rates of E = 100 and E = 5%. Click here for file

Additional file 2 **Appendix 2: Summary values for model results by testing parameter**. Model summary statistics (AUC, P, PSM) are reported for each category of testing parameter with error rates of E = 100 and E = 5%. Click here for file
