# Supplementary material for: Comparative analysis of remotely-sensed data products via ecological niche modeling of avian influenza case occurrences in Middle Eastern poultry
Source: Int J Health Geogr. 2011 Mar 28;10:21. doi: 10.1186/1476-072X-10-21 (PMC3078832; doi:10.1186/1476-072X-10-21)
Supplement: Additional file 1 — Appendix 1: Summary of models developed and partial ROC test results. Each of the 144 models developed are displayed along with associated AUC and P values for error rates of E = 100 and E = 5%. [file 1476-072X-10-21-S1.DOC]

Appendix 1. Summary of models developed and partial ROC test results in this study. AP = Arabian Peninsula, BC = Balkans and Caucasus, LI = Levant and Iran, AF = Northeast Africa*.* ”P” is the number of bootstrap receiver operating characteristic (ROC) iterations (out of 1000) in which the partial AUC value was < 1. *E* represents the expected/allowed error rate in the testing occurrence data in partial AUC analyses (Peterson et al. 2008), whereby E = 100 is equivalent to traditional ROC analysis, and E = 5% represents a partial AUC focused on models able to predict most or all of the independent testing points. Models showing bootstrap values less than 50 (one-tailed test, α = 0.05) fit data significantly better than expected and are signified with an *.

|  |  |  |  | *E* = 100 | | *E* = 5% | |
| --- | --- | --- | --- | --- | --- | --- | --- |
| Model Type | Data set | Training region | Testing region | AUC ratio | P | AUC ratio | P |
| **Random subsampling within single region** | | | | | | | |
| Monthly | LSWI | AF | AF | 1.5290 | 0* | 1.3658 | 0* |
| Monthly | EVI | AF | AF | 1.6428 | 0* | 1.3709 | 0* |
| Monthly | NDVI | AF | AF | 1.5987 | 0* | 1.3804 | 0* |
| Monthly | LSWI, EVI, NDVI | AF | AF | 1.6613 | 0* | 1.4391 | 0* |
| Monthly | LSWI | LI | LI | 0.75681 | 889 | 0.99866 | 629 |
| Monthly | EVI | LI | LI | 1.6081 | 4* | 1.1307 | 5* |
| Monthly | NDVI | LI | LI | 1.5462 | 36* | 1.0992 | 49* |
| Monthly | LSWI, EVI, NDVI | LI | LI | 1.2496 | 196 | 1.0122 | 185 |
| Monthly | LSWI | BC | BC | 1.1186 | 107 | 1.0071 | 215 |
| Monthly | EVI | BC | BC | 1.0199 | 423 | 1.0073 | 409 |
| Monthly | NDVI | BC | BC | 0.94536 | 755 | 1.0147 | 250 |
| Monthly | LSWI, EVI, NDVI | BC | BC | 0.99637 | 533 | 0.99613 | 684 |
| Monthly | LSWI | AP | AP | 1.1225 | 211 | 1.0224 | 269 |
| Monthly | EVI | AP | AP | 1.361 | 0* | 1.2326 | 0* |
| Monthly | NDVI | AP | AP | 1.2353 | 69 | 1.1091 | 0* |
| Monthly | LSWI, EVI, NDVI | AP | AP | 1.327 | 17* | 1.0701 | 14* |
| Summary | LSWI | AF | AF | 1.6444 | 0* | 1.3598 | 0* |
| Summary | EVI | AF | AF | 1.1568 | 0* | 1.0697 | 0* |
| Summary | NDVI | AF | AF | 1.5343 | 0* | 1.2076 | 0* |
| Summary | LSWI, EVI, NDVI | AF | AF | 1.6124 | 0* | 1.3838 | 0* |
| Summary | LSWI | LI | LI | 1.2031 | 255 | 1.0113 | 381 |
| Summary | EVI | LI | LI | 1.2443 | 204 | 1.0141 | 396 |
| Summary | NDVI | LI | LI | 1.6708 | 3* | 1.1268 | 3* |
| Summary | LSWI, EVI, NDVI | LI | LI | 1.2295 | 240 | 1.0155 | 81 |
| Summary | LSWI | BC | BC | 1.1284 | 88 | 1.0374 | 18* |
| Summary | EVI | BC | BC | 1.2642 | 2* | 1.0546 | 55 |
| Summary | NDVI | BC | BC | 1.1876 | 37* | 1.0306 | 0* |
| Summary | LSWI, EVI, NDVI | BC | BC | 1.2741 | 1* | 1.0011 | 444 |
| Summary | LSWI | AP | AP | 1.2979 | 81 | 1.0389 | 86 |
| Summary | EVI | AP | AP | 1.3452 | 7* | 1.2054 | 0* |
| Summary | NDVI | AP | AP | 1.3728 | 7* | 1.1953 | 0* |
| Summary | LSWI, EVI, NDVI | AP | AP | 1.4285 | 5* | 1.1362 | 12* |
| Monthly & summary | LSWI | AF | AF | 1.6267 | 0* | 1.4097 | 0* |
| Monthly & summary | EVI | AF | AF | 1.6810 | 0* | 1.4000 | 0* |
| Monthly & summary | NDVI | AF | AF | 1.6768 | 0* | 1.3792 | 0* |
| Monthly & summary | LSWI, EVI, NDVI | AF | AF | 1.6836 | 0* | 1.4253 | 0* |
| Monthly & summary | LSWI | LI | LI | 0.89656 | 642 | 0.99628 | 625 |
| Monthly & summary | EVI | LI | LI | 1.4755 | 35* | 1.0496 | 17* |
| Monthly & summary | NDVI | LI | LI | 1.5568 | 3* | 1.1222 | 4* |
| Monthly & summary | LSWI, EVI, NDVI | LI | LI | 1.1165 | 361 | 1.0049 | 359 |
| Monthly & summary | LSWI | BC | BC | 1.1754 | 37* | 1.0156 | 147 |
| Monthly & summary | EVI | BC | BC | 1.1461 | 46* | 1.0274 | 84 |
| Monthly & summary | NDVI | BC | BC | 1.0487 | 291 | 1.0109 | 173 |
| Monthly & summary | LSWI, EVI, NDVI | BC | BC | 1.0657 | 246 | 1.0006 | 466 |
| Monthly & summary | LSWI | AP | AP | 1.2795 | 58 | 1.0384 | 77 |
| Monthly & summary | EVI | AP | AP | 1.3348 | 17* | 1.1578 | 0* |
| Monthly & summary | NDVI | AP | AP | 1.5034 | 4* | 1.0578 | 69 |
| Monthly & summary | LSWI, EVI, NDVI | AP | AP | 1.3739 | 8* | 1.0849 | 2* |
| **Three regions predict one** | | | | | | | |
| Monthly | LSWI | BC, AF, LI | AP | 1.0925 | 97 | 1.0741 | 0* |
| Monthly | EVI | BC, AF, LI | AP | 1.2169 | 13* | 1.2158 | 0* |
| Monthly | NDVI | BC, AF, LI | AP | 1.1299 | 248 | 1.0100 | 0* |
| Monthly | LSWI, EVI, NDVI | BC, AF, LI | AP | 1.3628 | 0* | 1.2472 | 0* |
| Summary | LSWI | BC, AF, LI | AP | 1.2009 | 117 | 1.0620 | 0* |
| Summary | EVI | BC, AF, LI | AP | 1.2694 | 10* | 1.1396 | 0* |
| Summary | NDVI | BC, AF, LI | AP | 1.2439 | 18* | 1.1962 | 0* |
| Summary | LSWI, EVI, NDVI | BC, AF, LI | AP | 1.3872 | 0* | 1.2629 | 0* |
| Monthly & summary | LSWI | BC, AF, LI | AP | 1.1425 | 187 | 1.0734 | 0* |
| Monthly & summary | EVI | BC, AF, LI | AP | 0.98281 | 602 | 1.2141 | 0* |
| Monthly & summary | NDVI | BC, AF, LI | AP | 1.1822 | 70 | 1.1356 | 0* |
| Monthly & summary | LSWI, EVI, NDVI | BC, AF, LI | AP | 1.2333 | 31* | 1.1762 | 0* |
| Monthly | LSWI | AP, AF, LI | BC | 1.0413 | 136 | 1.0152 | 205 |
| Monthly | EVI | AP, AF, LI | BC | 1.0168 | 416 | 0.98431 | 563 |
| Monthly | NDVI | AP, AF, LI | BC | 0.88654 | 863 | 0.98252 | 840 |
| Monthly | LSWI, EVI, NDVI | AP, AF, LI | BC | 1.0125 | 437 | 0.9922 | 573 |
| Summary | LSWI | AP, AF, LI | BC | 0.97285 | 643 | 0.99965 | 273 |
| Summary | EVI | AP, AF, LI | BC | 1.1455 | 17* | 1.0099 | 230 |
| Summary | NDVI | AP, AF, LI | BC | 1.0746 | 168 | 1.0066 | 279 |
| Summary | LSWI, EVI, NDVI | AP, AF, LI | BC | 1.1113 | 85 | 1.0193 | 191 |
| Monthly & summary | LSWI | AP, AF, LI | BC | 0.99719 | 511 | 0.99853 | 271 |
| Monthly & summary | EVI | AP, AF, LI | BC | 0.94847 | 714 | 0.98665 | 491 |
| Monthly & summary | NDVI | AP, AF, LI | BC | 1.0171 | 423 | 1.0019 | 275 |
| Monthly & summary | LSWI, EVI, NDVI | AP, AF, LI | BC | 1.0041 | 484 | 0.98281 | 650 |
| Monthly | LSWI | AP, BC, LI | AF | 1.4703 | 0* | 1.0870 | 0* |
| Monthly | EVI | AP, BC, LI | AF | 1.1074 | 6* | 1.0308 | 1* |
| Monthly | NDVI | AP, BC, LI | AF | 0.94328 | 904 | 1.0172 | 85 |
| Monthly | LSWI, EVI, NDVI | AP, BC, LI | AF | 1.1560 | 6* | 1.0163 | 14* |
| Summary | LSWI | AP, BC, LI | AF | 1.1377 | 8* | 1.0070 | 18* |
| Summary | EVI | AP, BC, LI | AF | 1.0612 | 131 | 1.0053 | 44* |
| Summary | NDVI | AP, BC, LI | AF | 1.1103 | 31* | 1.0051 | 68 |
| Summary | LSWI, EVI, NDVI | AP, BC, LI | AF | 1.0740 | 90 | 1.0033 | 203 |
| Monthly & summary | LSWI | AP, BC, LI | AF | 1.2313 | 0* | 1.0074 | 152 |
| Monthly & summary | EVI | AP, BC, LI | AF | 0.95922 | 849 | 1.0359 | 0* |
| Monthly & summary | NDVI | AP, BC, LI | AF | 0.84879 | 1000 | 1.0128 | 103 |
| Monthly & summary | LSWI, EVI, NDVI | AP, BC, LI | AF | 1.1351 | 7* | 1.0143 | 17* |
| Monthly | LSWI | AP, BC, AF | LI | 0.75547 | 752 | 1.0244 | 0* |
| Monthly | EVI | AP, BC, AF | LI | 1.3954 | 63 | 1.1938 | 0* |
| Monthly | NDVI | AP, BC, AF | LI | 1.3841 | 2* | 1.2421 | 0* |
| Monthly | LSWI, EVI, NDVI | AP, BC, AF | LI | 1.3206 | 66 | 1.2107 | 0* |
| Summary | LSWI | AP, BC, AF | LI | 0.06017 | 836 | 0.09822 | 905 |
| Summary | EVI | AP, BC, AF | LI | 1.1314 | 181 | 1.0059 | 195 |
| Summary | NDVI | AP, BC, AF | LI | 1.5639 | 2* | 1.2649 | 0* |
| Summary | LSWI, EVI, NDVI | AP, BC, AF | LI | 1.0501 | 367 | 1.0063 | 439 |
| Monthly & summary | LSWI | AP, BC, AF | LI | 0.9376 | 628 | 1.0685 | 0* |
| Monthly & summary | EVI | AP, BC, AF | LI | 1.2619 | 0* | 1.3236 | 0* |
| Monthly & summary | NDVI | AP, BC, AF | LI | 1.3358 | 59 | 1.1822 | 0* |
| Monthly & summary | LSWI, EVI, NDVI | AP, BC, AF | LI | 1.0912 | 402 | 1.0061 | 404 |
| **One region predicts three** | | | | | | | |
| Monthly | LSWI | AP | BC, AF, LI | 1.0102 | 395 | 1.0613 | 0* |
| Monthly | EVI | AP | BC, AF, LI | 1.0627 | 31* | 1.0883 | 0* |
| Monthly | NDVI | AP | BC, AF, LI | 1.1511 | 0* | 1.0765 | 14* |
| Monthly | LSWI, EVI, NDVI | AP | BC, AF, LI | 0.93501 | 953 | 0.99807 | 913 |
| Summary | LSWI | AP | BC, AF, LI | 0.87203 | 999 | 1.003 | 361 |
| Summary | EVI | AP | BC, AF, LI | 1.1226 | 0* | 1.0876 | 0* |
| Summary | NDVI | AP | BC, AF, LI | 0.94335 | 926 | 1.0608 | 1* |
| Summary | LSWI, EVI, NDVI | AP | BC, AF, LI | 0.8821 | 997 | 0.099505 | 997 |
| Monthly & summary | LSWI | AP | BC, AF, LI | 1.0124 | 384 | 1.0529 | 0* |
| Monthly & summary | EVI | AP | BC, AF, LI | 1.1190 | 0* | 1.0998 | 0* |
| Monthly & summary | NDVI | AP | BC, AF, LI | 1.1869 | 0* | 1.0664 | 1* |
| Monthly & summary | LSWI, EVI, NDVI | AP | BC, AF, LI | 0.94277 | 925 | 0.99823 | 877 |
| Monthly | LSWI | BC | AP, AF, LI | 1.4014 | 0* | 1.0257 | 0* |
| Monthly | EVI | BC | AP, AF, LI | 0.94918 | 805 | 0.98148 | 1000 |
| Monthly | NDVI | BC | AP, AF, LI | 1.1892 | 0* | 1.0132 | 0* |
| Monthly | LSWI, EVI, NDVI | BC | AP, AF, LI | 1.2339 | 0* | 1.0103 | 0* |
| Summary | LSWI | BC | AP, AF, LI | 1.2844 | 0* | 1.0146 | 0* |
| Summary | EVI | BC | AP, AF, LI | 1.1011 | 5* | 0.99665 | 644 |
| Summary | NDVI | BC | AP, AF, LI | 1.2504 | 0* | 1.0189 | 0* |
| Summary | LSWI, EVI, NDVI | BC | AP, AF, LI | 1.1009 | 42* | 0.98823 | 997 |
| Monthly & summary | LSWI | BC | AP, AF, LI | 1.3963 | 0* | 1.0191 | 0* |
| Monthly & summary | EVI | BC | AP, AF, LI | 1.1426 | 0* | 1.0087 | 5* |
| Monthly & summary | NDVI | BC | AP, AF, LI | 1.1653 | 0* | 1.0107 | 0* |
| Monthly & summary | LSWI, EVI, NDVI | BC | AP, AF, LI | 1.2411 | 0* | 1.0113 | 0* |
| Monthly | LSWI | AF | AP, BC, LI | 1.046 | 145 | 0.99443 | 350 |
| Monthly | EVI | AF | AP, BC, LI | 0.89162 | 891 | 0.9810 | 972 |
| Monthly | NDVI | AF | AP, BC, LI | 0.91032 | 892 | 0.97814 | 873 |
| Monthly | LSWI, EVI, NDVI | AF | AP, BC, LI | 1.0820 | 97 | 1.0828 | 0* |
| Summary | LSWI | AF | AP, BC, LI | 0.71192 | 999 | 0.98917 | 816 |
| Summary | EVI | AF | AP, BC, LI | 1.2300 | 0* | 1.1482 | 0* |
| Summary | NDVI | AF | AP, BC, LI | 0.68011 | 1000 | 0.97127 | 968 |
| Summary | LSWI, EVI, NDVI | AF | AP, BC, LI | 0.77244 | 999 | 1.0012 | 389 |
| Monthly & summary | LSWI | AF | AP, BC, LI | 0.8499 | 978 | 1.0211 | 0* |
| Monthly & summary | EVI | AF | AP, BC, LI | 0.97382 | 618 | 0.98048 | 751 |
| Monthly & summary | NDVI | AF | AP, BC, LI | 0.67992 | 1000 | 0.98332 | 920 |
| Monthly & summary | LSWI, EVI, NDVI | AF | AP, BC, LI | 0.90488 | 944 | 1.0938 | 0* |
| Monthly | LSWI | LI | AP, BC, AF | 1.0957 | 15* | 1.0204 | 1* |
| Monthly | EVI | LI | AP, BC, AF | 0.99631 | 555 | 1.0005 | 349 |
| Monthly | NDVI | LI | AP, BC, AF | 1.0889 | 28* | 1.0119 | 0* |
| Monthly | LSWI, EVI, NDVI | LI | AP, BC, AF | 1.0205 | 322 | 1.0003 | 412 |
| Summary | LSWI | LI | AP, BC, AF | 1.3537 | 0* | 1.0149 | 2* |
| Summary | EVI | LI | AP, BC, AF | 1.1885 | 0* | 1.0077 | 0* |
| Summary | NDVI | LI | AP, BC, AF | 0.9967 | 552 | 0.99977 | 581 |
| Summary | LSWI, EVI, NDVI | LI | AP, BC, AF | 0.92152 | 965 | 0.99706 | 993 |
| Monthly & summary | LSWI | LI | AP, BC, AF | 1.2234 | 0* | 1.0213 | 0* |
| Monthly & summary | EVI | LI | AP, BC, AF | 0.96786 | 789 | 0.99932 | 651 |
| Monthly & summary | NDVI | LI | AP, BC, AF | 1.1235 | 4* | 1.0143 | 0* |
| Monthly & summary | LSWI, EVI, NDVI | LI | AP, BC, AF | 1.0167 | 303 | 1.0003 | 405 |
