# Supplementary material for: Comparative analysis of remotely-sensed data products via ecological niche modeling of avian influenza case occurrences in Middle Eastern poultry
Source: Int J Health Geogr. 2011 Mar 28;10:21. doi: 10.1186/1476-072X-10-21 (PMC3078832; doi:10.1186/1476-072X-10-21)
Supplement: Additional file 2 — Appendix 2: Summary values for model results by testing parameter. Model summary statistics (AUC, P, PSM) are reported for each category of testing parameter with error rates of E = 100 and E = 5%. [file 1476-072X-10-21-S2.DOC]

|  |  | E = 100 |  |  | E = 5% |  |
| --- | --- | --- | --- | --- | --- | --- |
| **Model Level** | AUC ratio | P | PSM | AUC ratio | P | PSM |
| Within one region | 1.332 | 123.292 | 0.625 | 1.132 | 129.333 | 0.563 |
| Three predict one | 1.108 | 264.167 | 0.354 | 1.055 | 156.021 | 0.563 |
| One predict three | 1.050 | 386.625 | 0.479 | 1.002 | 317.563 | 0.563 |
| **Index Category** |  |  |  |  |  |  |
| EVI | 1.181 | 206.500 | 0.611 | 1.090 | 190.611 | 0.611 |
| LSWI | 1.113 | 283.278 | 0.361 | 1.029 | 161.139 | 0.556 |
| NDVI | 1.193 | 260.972 | 0.556 | 1.083 | 154.333 | 0.639 |
| All three | 1.167 | 281.361 | 0.417 | 1.050 | 297.806 | 0.444 |
| **Index Type** |  |  |  |  |  |  |
| Monthly | 1.168 | 257.875 | 0.438 | 1.082 | 205.708 | 0.604 |
| Summary | 1.158 | 231.625 | 0.542 | 1.029 | 230.625 | 0.521 |
| Both | 1.164 | 284.583 | 0.479 | 1.079 | 166.583 | 0.583 |
| **Overall** | 1.164 | 258.028 | 0.486 | 1.063 | 200.972 | 0.563 |

Appendix 2: Summary values for model results by testing parameter. Values reported are averages for AUC ratio and P (bootstrap value). PSM is proportion of significant models, where significant models are those with P scores less than 50 (p = 0.05, one-way, 1000 bootstrap replicates. See Methods section for details.)
